# Functional Electrolytes: Game Changers for Smart Electrochemical Energy Storage Devices

**DOI:** 10.1002/smsc.202100080

**Published:** 2021-10-24

**Authors:** Faxing Wang, Panpan Zhang, Gang Wang, Ali Shaygan Nia, Minghao Yu, Xinliang Feng

**Affiliations:** ^1^ Center for Advancing Electronics Dresden (cfaed) & Faculty of Chemistry and Food Chemistry Technische Universität Dresden Mommsenstrasse 4 Dresden 01069 Germany

**Keywords:** functional electrolytes, rechargeable batteries, smart energy storage, stimulus‐responsive electrolytes, supercapacitors

## Abstract

Electrochemical energy storage (EES) devices integrated with smart functions are highly attractive for powering the next‐generation electronics in the coming era of artificial intelligence. In this regard, exploiting functional electrolytes represents a viable strategy to realize smart functions in EES devices. In this review, recent advances in the development of functional electrolytes for smart EES devices like rechargeable batteries and supercapacitors are presented. Various stimulus‐responsive electrolytes are first summarized, including temperature‐, mechanical force‐, voltage‐, magnetism‐, and light‐responsive electrolytes. Emphasis is placed on the working principles of stimulus‐responsive electrolytes and their specific problem‐solving functions in EES devices. Then, the latest advances in electrochromic and self‐healing electrolytes used for smart EES devices are discussed. Finally, key challenges and research opportunities related to functional electrolyte design and smart EES device development are envisioned, with the goal of accelerating further research in this thriving field.

## Graphical abstract

Smart electrochemical energy storage (EES) devices are highly attractive for powering next‐generation electronics in the coming era of artificial intelligence. In this review, the recent advances in the development of functional electrolytes for smart EES devices are introduced.

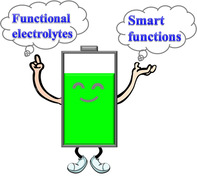

## 1 Introduction

The advance of artificial intelligence is very likely to trigger a new industrial revolution in the foreseeable future.^[^ ^1^, ^2^, ^3^ ^]^ Recently, the ever‐growing market of smart electronics is imposing a strong demand for the development of effective and efficient power sources. Electrochemical energy storage (EES) devices, including rechargeable batteries and supercapacitors, have been widely applied as power sources for portable electronics. Rechargeable batteries are usually used as electrochemical power sources for the requirement of large specific energy density.^[^ ^4^, ^5^ ^]^ Meanwhile, supercapacitors which feature high output power, exhibit complementary energy storage characteristics to rechargeable batteries.^[^ ^6^, ^7^, ^8^ ^]^ Beyond energy storage, future smart electronics would require their power sources to be more reliable under extreme or risky conditions. First, the internal conditions and parameters of EES devices (e.g., temperature, pressure, voltage, mechanical properties, and chemical environment) must be monitored in real time.^[^ ^9^ ^]^ Second, EES devices need to rapidly sense and regulate in response to external or internal environmental changes. However, conventional EES devices cannot meet these requirements.

**Figure 1:**
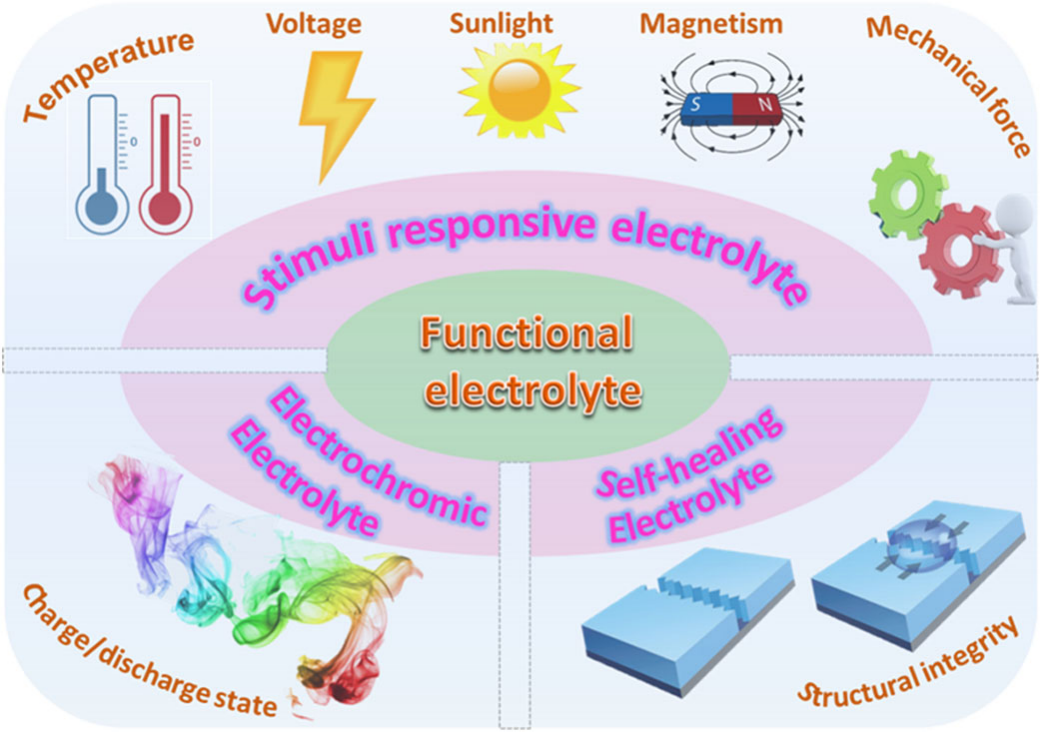
Schematic of the review content on functional electrolytes.

The incorporation of smart functionalities into EES devices with intrinsic abilities of real‐time monitoring, self‐protection, self‐charging, or self‐healing holds the key to addressing the aforementioned challenges. In this respect, considerable efforts have been devoted to developing smart EES devices with versatile functionalities, which are realized by delicately using functional electrodes, functional electrolytes, or functional current collectors.^[^ ^10^, ^11^, ^12^, ^13^, ^14^, ^15^, ^16^, ^17^, ^18^ ^]^ The designed smart functionalities in EES devices can spontaneously detect abnormalities at an early stage and trigger implementation of self‐protection or self‐adaptation. Among the various strategies for assembling smart EES devices, the integration of functional electrolytes into EES devices represents the most commonly used approach, as the electrolytes lie in the middle of EES devices and have easy access to all device components. Thus far, a variety of functional electrolytes have been reported for smart EES devices, showing sensitive response to various internal or external stimuli, such as temperature, mechanical deformation, voltage, light, magnetism, and structural integrity (**Figure** **1**).^[^ ^12^, ^13^, ^14^, ^15^, ^16^, ^17^, ^18^ ^]^ These smart electrolytes bestow fantastic capabilities on EES devices such as self‐protection, self‐power, performance improvement, prevention of electrolyte leakage, electrochromism, and self‐healing.

Although several comprehensive reviews have summarized the development of various electrolytes for EES devices,^[^ ^19^, ^20^, ^21^, ^22^, ^23^, ^24^ ^]^ a critical review specifically dedicated to functional electrolytes for smart EES devices is still lacking. Herein, we introduce the research frontier of functional electrolytes for EES devices. The first part is dedicated to provide the basic configurations of three types of functional electrolytes, including stimulus‐responsive electrolytes, electrochromic electrolytes, and self‐healing electrolytes. Then the significant advances of stimulus‐responsive electrolytes used for smart EES devices are summarized. They include i) temperature‐responsive electrolytes for overheat‐protection EES devices, ii) mechanical force‐responsive electrolytes for self‐powered EES devices, iii) abnormally high voltage‐responsive electrolytes for overcharge self‐protection EES devices, and v) magnetism and sunlight‐responsive electrolytes for electrolyte leakage prevention and performance enhancement of EES devices. Particular efforts are devoted to analyzing the working principles of functional electrolytes and their specific problem‐solving abilities for smart EES devices. Afterward, we discuss the novel electrochromic electrolytes for the real‐time display of the charging/discharging status of EES devices. Furthermore, we present the state‐of‐the‐art self‐healing electrolytes, which are based on reversible dynamic bond chemistries (e.g., hydrogen bonds and borate ester bonds) or electrostatic interactions. Finally, we provide our perspective on the remaining challenges and future research opportunities for the development of functional electrolytes and smart EES devices.

## 2 Basic Configurations of Functional Electrolytes

**Figure 2:**
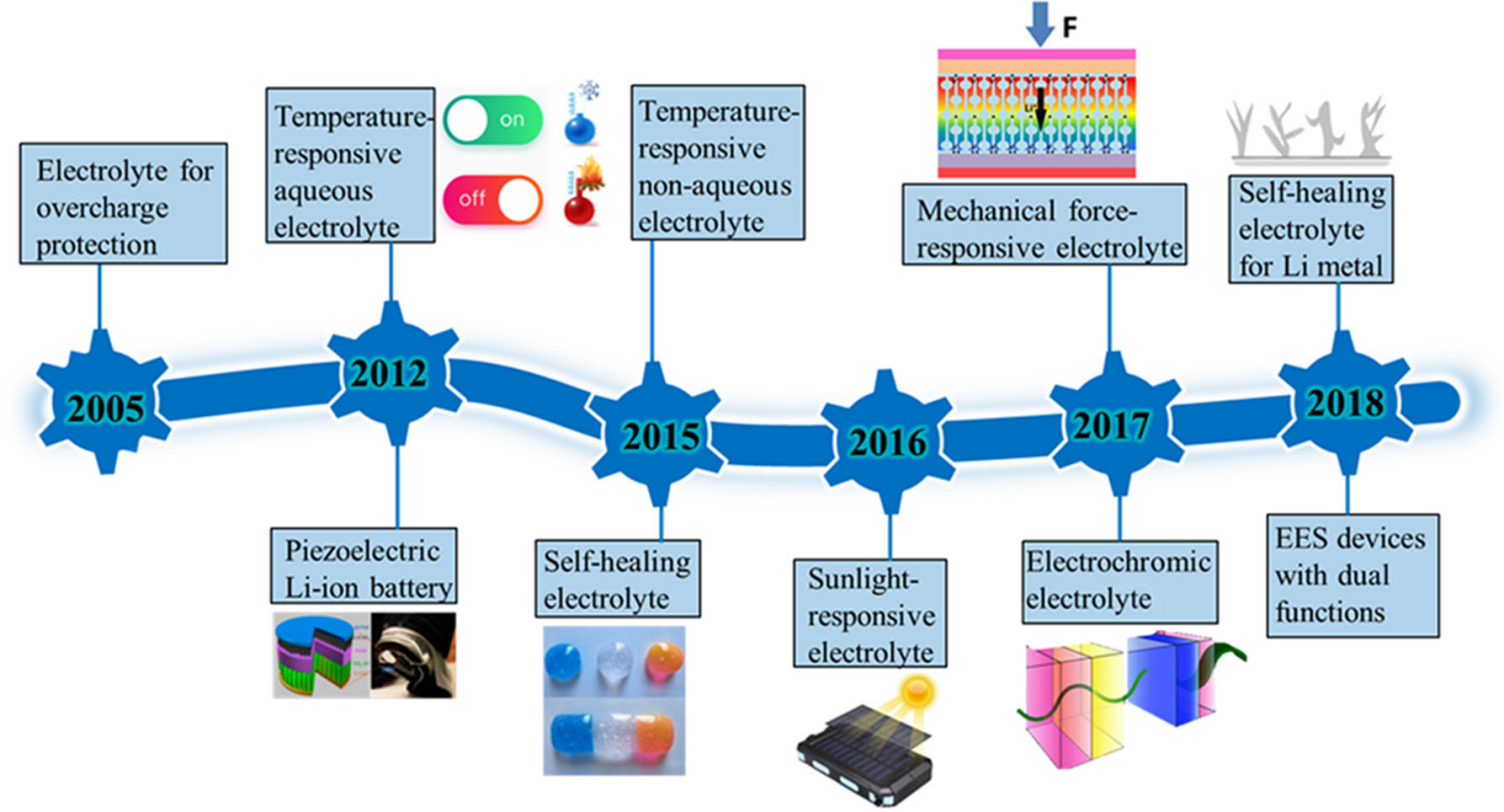
Brief development timeline of representative functional electrolytes and smart EES devices. Piezoelectric Li‐ion battery: Reproduced with permission.^[^ ^59^ ^]^ Copyright 2012, American Chemical Society. Self‐healing electrolyte: Reproduced with permission.^[^ ^115^ ^]^ Copyright 2016, Royal Society of Chemistry. Mechanical force‐responsive electrolyte: Reproduced with permission.^[^ ^63^ ^]^ Copyright 2017, Elsevier Ltd. Electrochromic electrolyte: Reproduced with permission.^[^ ^99^ ^]^ Copyright 2016, Elsevier Ltd.

Functional electrolytes are typically composed of electrolyte salts, solvents, and functional species. The key components are the functional species that offer specific smart functions to EES devices. Aside from the specific smart functions, ideal functional electrolytes should possess the following characteristics: i) high chemical and electrochemical inertness to other EES device components (e.g., electrodes, current collectors, and packaging); ii) large ion conductivities and transference numbers of active ions; iii) suitable responsive time; iv) wide electrochemical potential windows; and v) low processing cost. Typically, according to the function principles, functional electrolytes can be divided into three categories, namely stimulus‐responsive electrolytes, electrochromic electrolytes, and self‐healing electrolytes. The rapid development of functional electrolytes for smart EES devices has taken place mainly in the past decade (**Figure** **2**).

Early studies on stimulus‐responsive electrolytes were mainly focused on voltage‐responsive electrolytes containing redox‐shuttle additives, which were used to prevent the overcharge abuse of Li‐ion batteries.^[^ ^17^ ^]^ Like overcharging abuse, thermal runaway is another severe safety concern of EES devices. Temperature‐responsive electrolytes were explored to avoid the thermal runaway of EES devices.^[^ ^13^ ^]^ Typically, temperature‐responsive electrolytes can efficiently inhibit the ionic or electron conduction between the anode and the cathode at overheating states, thereby switching off the EES devices. Apart from self‐protection functions, the self‐powered function is attractive for EES devices. Direct collection and conversion of mechanical energy into electric energy for storage can be realized in self‐powered EES devices with mechanical force‐responsive electrolytes.^[^ ^12^, ^18^ ^]^ In addition, magnetism‐ and sunlight‐responsive electrolytes were demonstrated to prevent electrolyte leakage and enhance the electrochemical performance of EES devices.

Another two types of functional electrolytes are electrochromic electrolytes and self‐healing electrolytes.^[^ ^12^, ^15^, ^18^ ^]^ Electrolytes integrated with electrochromism are desirable because the color change induced by the applied voltage enables the real‐time display of the charging state of EES devices. It is worth noting that self‐healing electrolytes are more common than electrochromic electrolytes for applications in various EES devices. The development of self‐healing electrolytes was inspired by the natural self‐healing capability of tissue and skin. The self‐healing electrolytes can effectively repair cracks and fractures of EES devices, affording reliable applications for flexible/stretchable electronics.

## 3 Stimulus‐Responsive Electrolytes for EES

Stimulus response refers to the sensing and adaption to certain triggering from the surrounding environment. Stimulus‐responsive electrolytes can alter their chemical and/or physical properties upon exposure to external stimuli, rendering the EES devices with diverse smart functions. Introducing stimulus‐responsive functions into electrolytes shows great potential to revolutionize EES devices for future smart electronics. Currently, the most eye‐catching stimulus‐responsive electrolytes include temperature‐responsive, mechanical force‐responsive, voltage‐responsive, magnetism‐responsive, and sunlight‐responsive electrolytes.

### 3.1 Temperature‐Responsive Electrolytes for Overheating‐Protection EES Devices

The thermal runaway of EES devices, such as maloperation under high temperature and overheating induced by the external or internal short circuit, can trigger spontaneous exothermic reactions that may eventually lead to fire or explosion problems of EES devices using organic electrolytes. Although the thermal runaway of EES devices using aqueous electrolytes would not pose a fire or explosion hazard, internally accumulated heat would shorten the operation lifetime of EES devices and cause device failure due to the accelerated electrolyte volatilization. Currently, three overheating‐protection approaches have been proposed to guarantee safe and stable EES devices under a high‐temperature environment. The first strategy is switching off the electron transport in EES devices using temperature‐responsive electrodes or current collectors.^[^ ^25^, ^26^ ^]^ This approach needs to introduce extra materials with positive‐temperature coefficients to conventional electrodes or current collectors, which inevitably sacrifice specific energy/power densities of EES devices. The second approach is to inhibit the ion diffusion between electrodes using thermal‐shutdown separators.^[^ ^27^, ^28^ ^]^ The thermal shutdown separators are built based on polymer microspheres with melting points in the range of 80–180 °C. Once the internal temperature of EES devices reaches the polymer melting point, the solid polymer microspheres will turn into a fused liquid and be covered on the electrode surface, blocking the ion diffusion, and switching off the EES devices. However, such thermal response of thermal‐shutdown separator is irreversible. By contrast, using temperature‐responsive electrolytes in EES devices represents the third approach,^[^ ^13^, ^29^, ^30^ ^]^ which can avoid the sacrifice of energy/power densities associated with the introduction of inactive materials into electrodes. In general, temperature‐responsive electrolytes protect EES devices from overheating by reversible phase transition, reversible phase separation, and irreversible releasing of flame‐retardant additives.^[^ ^29^ ^]^ Specifically, temperature‐responsive electrolytes based on the phase transition or phase separation mechanisms leads to great changes in the interior microstructures of electrolytes or electrode surfaces at high temperatures, such as the ion movement ability of electrolytes and a non‐conductive layer of the electrode surface. Meanwhile, temperature‐responsive electrolytes with flame retardant‐polymer core–shell configurations are also developed. In such electrolytes, the polymer shell can be melted at high temperatures, releasing flame retardant to prevent the fire risk of EES devices. In this section, we summarize the recent advances in temperature‐responsive electrolytes used for constructing overheating‐protection EES devices.

#### 3.1.1 Temperature‐Responsive Electrolytes with Reversible Phase Transition

At room temperature, temperature‐responsive electrolytes with reversible phase transition are in liquid states, allowing electrolyte ions to move smoothly without restriction. When the temperature increases, phase transition of the electrolyte from liquid states to gel (or solid) states occurs, which would suppress the free movement of the electrolyte ions. Currently, the most used temperature‐responsive electrolytes are based on poly(*N*‐isopropylacrylamide) (PNIPAM).^[^ ^31^, ^32^, ^33^, ^34^, ^35^, ^36^, ^37^ ^]^ As a typical temperature‐responsive polymer, PNIPAM has a lower critical solution temperature (LCST) of around 32 °C. LCST is the critical temperature below which all compositions of the electrolyte are miscible in liquid states. Once the temperature increases above LCST, PNIPAM would experience a fast and reversible phase transition from a swollen, hydrophilic, and liquid state to a shrunken, hydrophobic, and solid state. In the solid state, the free movement of polymer chains and their surrounding ions would be inhibited. However, the low LCST cannot allow PNIPAM‐based electrolytes to be directly used for thermal‐switching EES devices. To this end, the LCST of PNIPAM‐based electrolytes were adjusted by functionalizing the chain ends of copolymers and altering the concentration of electrolyte salts. Various PNIPAM‐based copolymers with different chain‐ends have been applied in temperature‐responsive electrolytes with relatively high LCST for thermal‐switching EES (**Table** **1**), such as poly(*N*‐isopropylacrylamide‐*co*‐acrylic acid) (PNIPAM/AA) for pseudocapacitors,^[^ ^31^ ^]^ poly(*N*‐isopropylacrylamide‐*co*‐2‐acrylamido‐2‐methyl propane sulfonic acid) (PNIPAM/SPMA) for supercapacitors,^[^ ^32^ ^]^ poly(*N*‐isopropylacrylamide‐*co*‐acrylamide) (PNIPAM/AM) for supercapacitors and Zn‐ion batteries,^[^ ^33^, ^34^, ^35^ ^]^ poly(*N*‐isopropylacrylamide‐*co*‐methylcellulose) (PNIPAM/MC) for microsupercapacitors and sodium–bromine (Na–Br_2_) batteries.^[^ ^36^, ^37^ ^]^

**Table 1:** Comparisons of EES devices with the overheating‐protection function based on PNIPAM copolymers

EES device	Electrolyte	LCST	Switch off capacity ratio	Switch on/off cycles	Ref.
Symmetric supercapacitors using PANI	PNIPAM/AA/H_2_SO_4_	50 °C	85%	4	[31]
Microsupercapacitor using CNT/AC/GO	Pluronic/PNIPAM/AM/H_3_PO_4_	60–80 °C	>90%	6	[33]
Symmetric supercapacitor using CNT	PNIPAM/AM/LiOH	47 °C	65%	4	[34]
Zn‐ion battery based on Zn||PANI	PNIPAM/AM/Zn(CF_3_SO_3_)_2_	40–50 °C	>99%	5	[35]
Microsupercapacitor using PEDOT	PNIPAM/MC/LiCl	70 °C	>99%	5	[36]
Aqueous/nonaqueous Na||Br_2_ battery	PNIPAM/MC/NaBr	50–65 °C	95%	2	[37]

**Figure 3:**
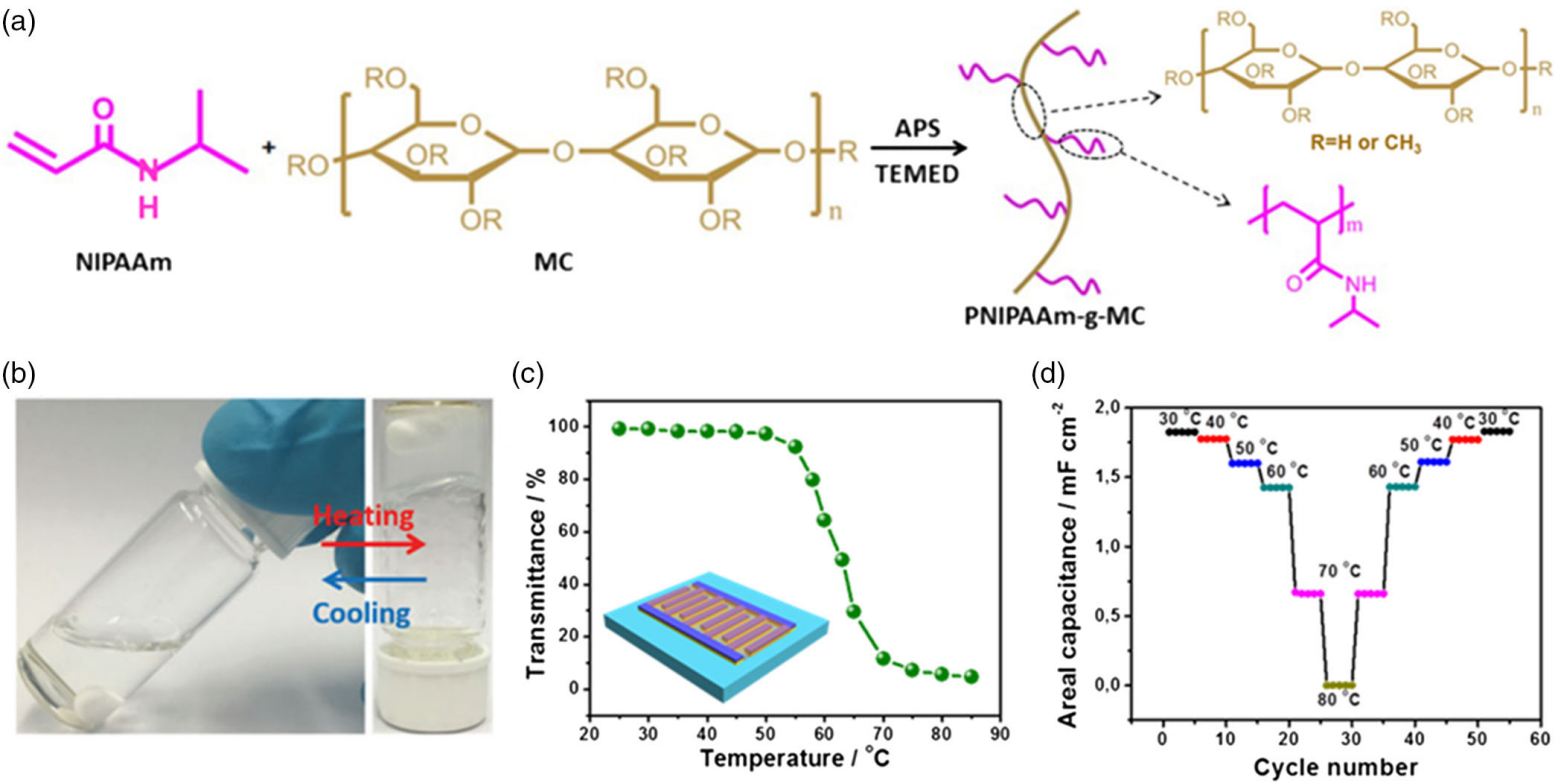
Temperature‐responsive electrolyte with reversible phase transition for microsupercapacitors. a) Synthesis route of the temperature‐responsive PNIPAAm/MC copolymer. b) Digital photographs of the reversible sol–gel transition of the PNIPAAm/MC solution at 30 and 80 °C. c) Temperature‐dependent transmittance of the PNIPAAm/MC solution. The inset is the schematic illustration of the assembled microsupercapacitor with the PNIPAAm/MC/LiCl electrolyte. d) Areal capacitances of the microsupercapacitors at different operating temperatures from 30 to 80 °C. a–d)Reproduced with permission.^[^ ^36^ ^]^ Copyright 2018, The Royal Society of Chemistry.

Among PNIPAM‐based temperature‐responsive polymers, PNIPAM/MC and Pluronic/PNIPAM/AM exhibit the highest LCSTs in the range of 65–80 °C. Recently, our group demonstrated PNIPAM/MC in the LiCl solution (PNIPAM/MC/LiCl) as an interesting temperature‐responsive electrolyte.^[^ ^36^ ^]^ PNIPAAm/MC was synthesized through free radical polymerization using *N*‐isopropylacrylamide (NIPAM) grafted onto methylcellulose (MC) as the monomer (**Figure** **3**a). During the polymerization reaction, ammonium persulfate (APS) and *N*,*N*,*N*′,*N*′‐tetramethylethylenediamine (TEMED) were used as an initiator and accelerator, repetitively. As shown in Figure 3b, PNIPAM/MC was dissolved in the 1 m LiCl aqueous electrolyte. The hydrophilic *N*‐isopropyl groups formed hydrogen bonds at the side chains of PNIPAM, allowing the free movement of ions in the obtained functional electrolyte. Once the temperature exceeded the LCST, the intermolecular interactions in MC backbones produced hydrophobic junctions and broke the hydrogen bonds. Thus, the PNIPAAm/MC electrolyte transformed immediately from a transparent liquid state to a white gel state, which was verified by the decrease in the transmittance of UV–vis spectra (Figure 3c). The ionic conductivity of the PNIPAAm/MC/LiCl electrolyte displayed a three orders of magnitude decay from the sol state (10 mS cm^−1^) to the gel state (0.034 mS cm^−1^).^[^ ^36^ ^]^ Moreover, a microsupercapacitor was assembled using poly(3,4‐ethylenedioxythiophene) interdigital electrodes and the PNIPAAm/MC/LiCl electrolyte. The as‐fabricated microsupercapacitor based on poly(3,4‐ethylenedioxythiophene)(PEDOT) electrodes exhibited a sharp decrease in the areal capacitance from 1.83 mF cm^−2^ at 30 °C to 0.66 mF cm^−2^ at 70 °C. Notably, the microsupercapacitor was completely switched off at 80 °C (Figure 3d), demonstrating the effective restriction of ion migration in the PNIPAAm/MC matrix.

Likely, the PNIPAM/MC/NaBr solution was used as the catholyte for high‐energy Na–Br_2_ batteries, and the overall capacity of Na–Br_2_ batteries decreased by 95% with the increasing temperature from 25 to 65 °C.^[^ ^37^ ^]^ Due to the toxicity and low boiling point of bromine in solution, the gradual volatilization of bromine in metal–Br_2_ batteries at high temperatures could be harmful to the user's health and cause the loss of active cathode materials. It is worth noting that the formation of gel at 65 °C would extremely retard the volatilization of bromine, which suppresses the active material loss and improve the safety issue of Na–Br_2_ battery when used at high temperatures. More importantly, the normal charge/discharge performance of EES devices using PNIPAM‐based electrolytes could recover to the initial states due to the reversible sol–gel transition of PNIPAM upon heating/cooling processes.

Apart from PNIPAM‐based copolymers, pluronic, poly(ethylene oxide)‐block‐poly(propylene oxide)‐block‐poly(ethylene oxide) (denoted PEO–PPO–PEO), also shows reversible sol–gel transition in H^+^‐ or Li^+^‐containing aqueous solutions.^[^ ^38^ ^]^ In PEO–PPO–PEO, the PEO segments are hydrophilic at room temperature, while they turn to be hydrophobic in the form of micellar structure. In this respect, PEO–PPO–PEO can be dissolved in water with a liquid form at room temperature, enabling ions to move freely across the electrolyte. Upon increasing the temperature, the formation of closely packed spherical micelles and the entanglement of stretched PEO segments would cause the transformation of PEO–PPO–PEO solution from the flowing liquid state to the nonflowing gel state. At the gel state, the steric effect of stretched polymer chains and the entangled polymer micelles greatly inhibit the motion of electrolyte ions. Therefore, the fabricated supercapacitors using PEO–PPO–PEO‐based electrolyte exhibited nearly 100% decay in the specific capacitance when the temperature increased from 20 to 70 °C.^[^ ^38^ ^]^ Although EES devices using both PEO–PPO–PEO and PNIPAM‐based electrolytes realized 100% switch off ratio of capacitance at above LSCT, switch on/off cycles of all the above EES devices at cooling/heating states are still unsatisfying and less than ten cycles.

#### 3.1.2 Temperature‐Responsive Electrolytes with Reversible Phase Separation

Thus far, temperature‐responsive polymers with reversible phase transition were only demonstrated in aqueous electrolytes. Their use in constructing nonaqueous EES devices is still restricted. Different from the sol–gel phase transition mechanism, the phase separation mechanism enables temperature‐responsive polymers to separate from the organic or ionic liquid (IL) electrolytes when the temperature increases to the LCST. Such temperature‐responsive polymers can form barrier layers at the electrode/electrolyte interface to shut down the charge transfer and switch off EES devices. Currently, several temperature‐responsive electrolytes with the reversible phase separation mechanism have been developed, including PEO/LiBF_4_/IL,^[^ ^39^ ^]^ poly(benzyl methacrylate) (PBMA)/LiTFSI/IL,^[^ ^40^ ^]^ and vinylene carbonate (VC)/LiI/LiPF_6_/carbonate.^[^ ^41^ ^]^

**Figure 4:**
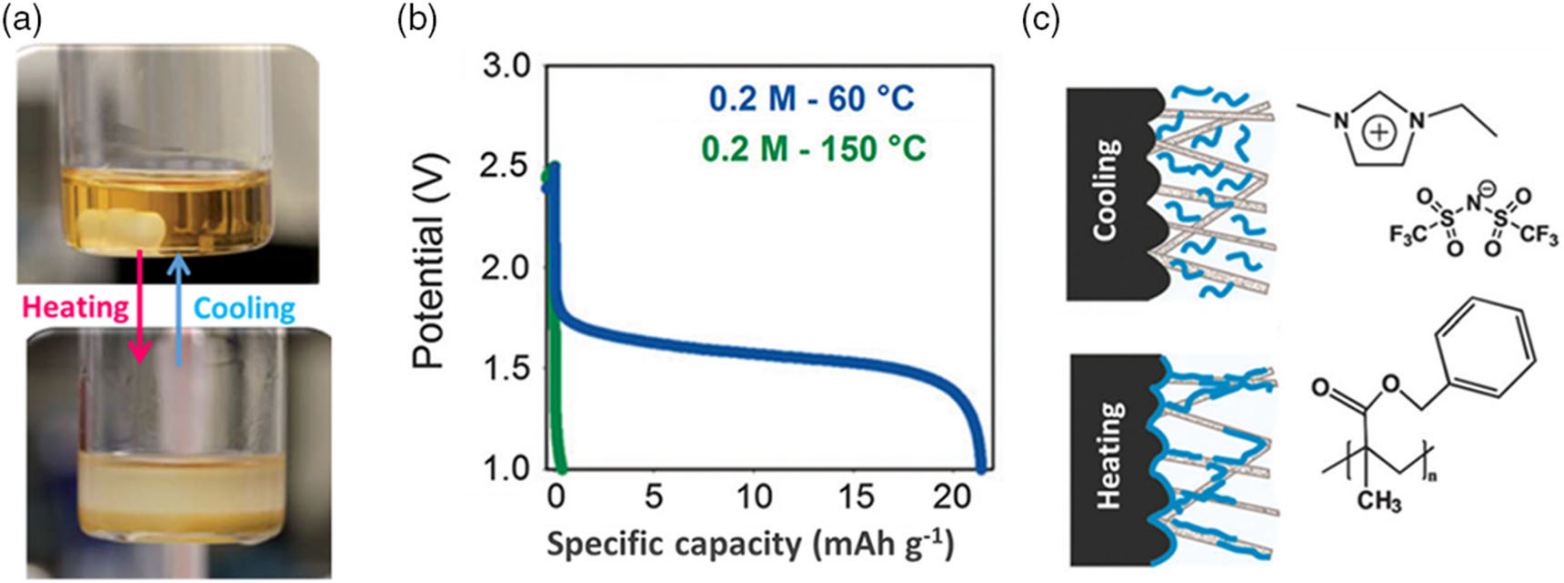
Temperature‐responsive electrolyte with reversible phase separation for Li‐ion batteries. a) Digital photographs showing the phase separation of the PEO/[EMIM][BF_4_] solution at heating and cooling states. Reproduced with permission.^[^ ^39^ ^]^ Copyright 2015, The Royal Society of Chemistry. b) The discharge profiles of the assembled Li_4_Ti_5_O_12_||LiFePO_4_ batteries in the PBMA/LiTFSI/[EMIM][TFSI] electrolyte with 0.2 m LiTFSI at 60 and 150 °C. c) Schematic of the structure of PBMA and IL, as well as the thermal‐switching mechanism at the electrode/electrolyte interface. b,c) Reproduced with permission.^[^ ^40^ ^]^ Copyright 2015, The Royal Society of Chemistry.

In 2015, Roberts and coworkers^[^ ^39^, ^40^ ^]^ pioneered the study on the thermal‐switching PEO/LiBF_4_/IL and PBMA/LiTFSI/IL electrolytes. Although PEO has been extensively applied as a polymer matrix for LiPF_6_‐ and LiTFSI‐based electrolytes without showing the temperature‐responsive behavior,^[^ ^42^, ^43^, ^44^ ^]^ PEO combining with [EMIM][BF_4_] ionic liquid and LiBF_4_ salt displayed remarkable thermal‐switching function.^[^ ^39^ ^]^ The PEO/[EMIM][BF_4_] solution showed a high ionic conductivity (10^−2^ S cm^−1^) because of its good fluidity at room temperature. After heating to above 125 °C, the PEO/[EMIM][BF_4_] solution was separated into a poorly conducting PEO‐rich solid phase and a well‐conducting IL‐rich liquid phase (**Figure** **4**a). The ionic conductivity of the PEO/[EMIM][BF_4_] electrolyte at the liquid–solid separating state decreased by an order of magnitude. Moreover, the LCST of the PEO/[EMIM][BF_4_] solution can be readily tailored by adding the LiBF_4_ salt or altering the concentration of LiBF_4_ salt.^[^ ^39^ ^]^ Apart from PEO, PBMA with an LCST of 100–140 °C exhibited a similar liquid–solid phase separation in the imidazolium‐based IL.^[^ ^45^ ^]^

Furthermore, the temperature‐responsive PBMA/LiTFSI/[EMIM][TFSI] electrolyte was applied to switch off Li‐ion batteries at abnormally high temperatures.^[^ ^40^ ^]^ The thermal‐switching Li‐ion batteries were assembled based on the Li_4_Ti_5_O_12_ anodes and LiFePO_4_ cathodes in the PBMA/LiTFSI/[EMIM][TFSI] electrolyte with 0.2 m LiTFSI. The discharge capacity of the assembled Li‐ion batteries decreased from 20 to near 0 mAh g^−1^, when the operating temperature increased from 60 to 150 °C (Figure 4b). At temperature above the LCST, the precipitated PMBA formed insulating coating layers on the Li_4_Ti_5_O_12_ and LiFePO_4_ electrodes (Figure 4c), consequently increasing the charge transfer resistance of electrodes and switching off the Li_4_Ti_5_O_12_||LiFePO_4_ batteries. Interestingly, a small difference was observed between the PEO/LiBF_4_/IL and PBMA/LiTFSI/[EMIM][TFSI] electrolytes. Specifically, the phase separation of PEO caused a decrease in the ionic conductivity of the electrolyte. However, PEO was still capable of coordinating and transporting Li^+^ above the LCST. By contrast, the precipitated PBMA during the phase separation process worked as an electronic insulator and could not transport Li^+^ above the LCST.

In addition, the VC/LiI/LiPF_6_/carbonate electrolyte was recently demonstrated as an effective temperature‐responsive electrolyte.^[^ ^41^ ^]^ In this electrolyte, LiI worked as both the electrolyte salt and the catalyst for the polymerization of VC at high operating temperatures. The VC polymerization rate using the LiI catalyst showed high dependence on the temperature. When the LiI/VC electrolyte was sealed in a coin cell, the polymerization rate was very slow at temperatures below 60 °C. Thus, the VC/LiI/LiPF_6_/carbonate electrolyte was in a gel form at room temperature, presenting a high ionic conductivity of 1.8 mS cm^−1^. Moreover, the reduction of trace VC on the surface of Li metal formed a robust solid electrolyte interphase (SEI) layer, which was beneficial for the uniform Li plating/stripping. When the assembled Li‐metal batteries were heated to 80 °C, the polymerization of VC was greatly accelerated, producing a large amount of solid poly(vinylene carbonate) precipitation. Finally, the gel electrolyte turned into the solid phase due to the complete polymerization of VC. The produced poly(vinylene carbonate) precipitation led to the sharp increase in the electrolyte resistance by a factor of 1000, thereby completely switching off the Li‐metal batteries.^[^ ^41^ ^]^ It should be pointed out that the thermal‐activated VC polymerization was irreversible. Consequently, Li‐metal batteries using the VC/LiI/LiPF_6_/carbonate electrolyte failed to restore the normal charging/discharging performance when the temperature recovered back to room temperature.

#### 3.1.3 Temperature‐Responsive Electrolytes with Flame‐Retardant Additives

Due to the high flammability, organic liquid electrolytes are believed to be the main reason for fires and explosions in nonaqueous EES devices. Directly adding flame‐retardant additives into conventional organic liquid electrolytes is an effective approach to prevent the fire risk of electrolytes at high temperatures. Currently, organic phosphorus compounds and their halogenated derivate have been widely explored as flame‐retardant additives for Li‐ion batteries.^[^ ^46^ ^]^ These flame‐retardant additives hinder the electrolyte burning by chemical radical‐scavenging process. At abnormally high temperatures, the flame‐retardant additive thermally decomposes into phosphorus‐containing free radical species, which can bind and terminate the hydrogen‐ and hydroxide‐free radicals generated during the combustion. Although flame‐retardant additives in electrolytes can significantly decrease the flammability risk at high temperatures, they would induce side reactions on the electrodes or increase the viscosity of the electrolyte, which sacrifices the stability of the electrode‐electrolyte interfaces and ionic conductivities of electrolytes.^[^ ^47^ ^]^ To solve this problem, flame‐retardant polymer core–shell additives were delicately designed, in which flame retardants as the core were encapsulated by the temperature‐responsive polymer shell.^[^ ^48^, ^49^, ^50^, ^51^, ^52^, ^53^, ^54^ ^]^ Certainly, the temperature‐responsive polymer shell should have good compatibility with the electrolytes and electrode materials. Up to now, the used temperature‐responsive polymer shells include poly(methyl methacrylate) (PMMA),^[^ ^48^ ^]^ poly(urea‐formaldehyde) (PUF),^[^ ^49^, ^50^, ^51^, ^52^ ^]^ poly(vinylidene fluoride‐hexafluoropropylene) (PVDF‐HFP),^[^ ^53^ ^]^ and poly(VC).^[^ ^54^ ^]^ At normal operating temperatures, the encapsulation of flame‐retardant additives by these polymers can prevent the direct exposure of flame‐retardant additives to the electrolytes, effectively overcoming the poor miscibility between flame‐retardant additives and electrolytes. Temperature‐responsive polymer shells would be melted when the temperature increases to their melting points. As a result, the encapsulated flame‐retardant additives are released into the electrolytes, extinguishing the fire of the highly flammable electrolytes. Such core–shell composites were also named “self‐extinguishing electrolyte additives.” Recently, the standard nail penetration test of the fully charged Li‐ion batteries with an overall capacity of 500 mAh showed that the use of the self‐extinguishing electrolyte additive suppressed the temperature rise in the nail test by 74%.^[^ ^48^ ^]^ Moreover, the flames of the electrolyte were completely extinguished within 1 s.^[^ ^53^ ^]^

Apart from liquid electrolytes, the self‐extinguishing strategy was also utilized for the solid‐liquid hybrid electrolytes.^[^ ^54^ ^]^ Triethyl phosphate (TEP) as the flame‐retardant additive was encapsulated into a robust solid poly(VC) matrix. The gelation process led to the strong interactions among polymer, TEP, and Li salt, inhibiting the undesired parasitic reactions between TEP and Li metal at the electrode/electrolyte interface. The Young's modulus of the solid‐liquid hybrid electrolyte with the poly(VC)/TEP additive reached up to 12.4 GPa, which was sufficiently high for preventing the propagation of Li dendrite. Impressively, the fabricated Li||Li symmetric batteries were stably operated for more than 500 h with only a small overpotential of 0.07 V. Moreover, the Ah‐level Li||LiNi_0.8_Co_0.1_Mn_0.1_O_2_ batteries were demonstrated to show enhanced thermal stability against thermal abuse, which had no sign of smoking or burning under the nail test by the authorized third party.^[^ ^54^ ^]^

### 3.2 Mechanical Force‐Responsive Electrolytes for Self‐Powered EES Devices

In sustainable energy exploitation, energy generation and energy storage are two important technologies requiring distinctive devices. Energy generation devices convert the original forms of energy (e.g., thermal energy, mechanical energy, and solar energy) into electricity, and energy storage devices convert electricity into chemical energy. Separated energy generation and storage processes inevitably cause partial energy loss and low energy utilization efficiency. Recent studies on piezoelectric EES devices demonstrated that energy could be generated and stored with a single device that converted mechanical energy directly into electrochemical energy. Integrating energy generation and energy storage into a single device bypassed the intermediate step of electricity generation and reduced the energy waste in the rectifying circuit.^[^ ^55^, ^56^, ^57^ ^]^ One straightforward strategy for assembling piezoelectric EES devices is using the polarized PVDF film to replace the traditional separators (e.g., polypropylene film or polyethylene film) in EES devices. The piezoelectricity is associated with the dipole moments and orientation of the polarized PVDF film. The higher electronegativity of fluorine atoms than those of carbon and hydrogen atoms triggers the excellent piezoelectric response.^[^ ^58^ ^]^ In 2012, Wang and coworkers,^[^ ^59^ ^]^ for the first time, reported a piezoelectric Li‐ion battery using the polarized PVDF film as the separator in a liquid electrolyte of 1 m LiPF_6_ dissolved in ethylene carbonate/dimethyl carbonate. However, the used liquid electrolyte is one of the obstacles for the practical application due to the electrolyte leakage problem upon applying mechanical deformation. Moreover, the fluidity of liquid electrolytes reduces the electromechanical conversion efficiency.

To address the electrolyte leakage issue, solid‐state polymer electrolytes with the mechanical force‐responsive capability were developed for piezoelectric EES devices.^[^ ^60^, ^61^, ^62^ ^]^ For example, the polarized PVDF/ZnO and natural bioseparators were integrated into the polyvinyl alcohol (PVA) matrix, serving as the solid electrolytes and piezoseparators for piezoelectric supercapacitors.^[^ ^61^, ^62^ ^]^ The assembled supercapacitors could be charged up to 0.28 V from its initial open‐circuit voltage (0.13 V) in 80 s by the continuous mechanical force from the human finger. As the proof‐of‐the concept, the assembled supercapacitors were attached to the bottom of a shoe, and walking could generate enough compressive energy to charge the supercapacitors.^[^ ^62^ ^]^

**Figure 5:**
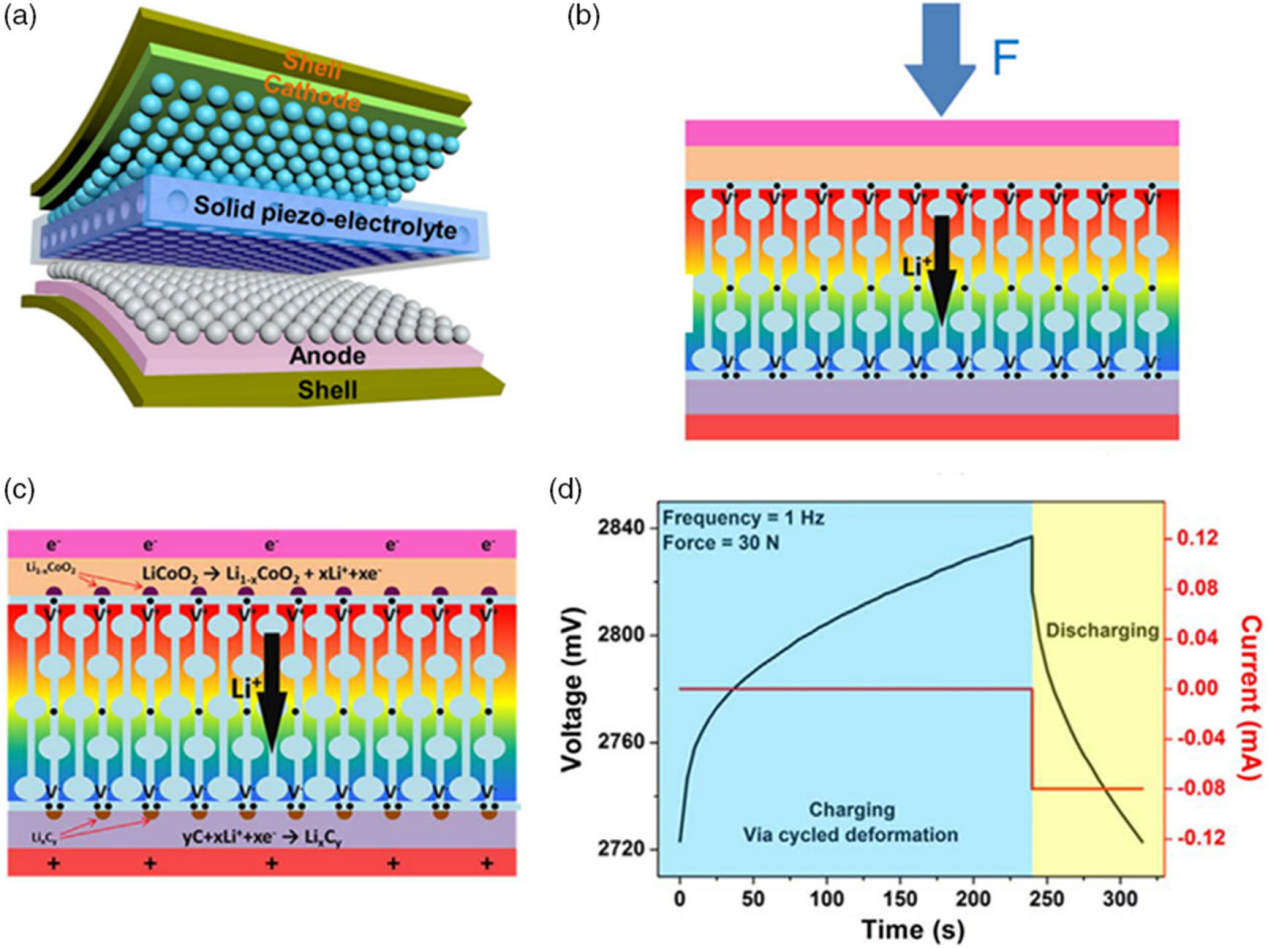
Mechanical force‐responsive electrolyte for self‐powered Li‐ion batteries. a) Schematic illustrating the structure of the assembled Li‐ion batteries. b,c) Schematic showing the piezoelectric mechanism of the assembled Li‐ion batteries. d) Self‐charging curve and subsequent discharge curve of the assembled Li‐ion batteries. a–d)Reproduced with permission.^[^ ^63^ ^]^ Copyright 2017, Elsevier Ltd.

In addition, self‐powered Li‐ion batteries (**Figure** **5**a) were fabricated with solid‐state polymer electrolytes based on the mesoporous PVDF‐LiPF_6_ film.^[^ ^63^ ^]^ Under a compressive mechanical force, the piezoelectric potential from the polarized PVDF film drove Li^+^ to migrate from the cathode to the anode. This process was accompanied by the oxidation reaction forming Li_1−*x*_CoO_2_ at the cathode and the reduction reaction inducing Li^+^ intercalation into the graphite anode (Figure 5b). Li^+^ would not flow back immediately when the applied mechanical force was removed because both the anode and cathode formed stable charging compounds (Figure 5c). The assembled graphite||LiCoO_2_ batteries were charged under a compressive deformation (30 N, 1 Hz) for 240 s, and the storage capacity achieved 1.67 μAh (Figure 5d). The mesoporous structure of the polarized PVDF film ensured the high piezoelectric output and ionic conduction paths for the Li^+^ migration.^[^ ^63^ ^]^ To further enhance the piezoelectric potential, several inorganic additives were dispersed into the polarized PVDF matrix to form solid composite electrolytes, such as Pb(Zr_*x*_Ti_1−*x*_)O_3_ and 0.5(Ba_0.7_Ca_0.3_)TiO_3_‐0.5Ba(Zr_0.2_Ti_0.8_)O_3_.^[^ ^64^, ^65^ ^]^ These inorganic piezoelectric additives can further enhance the piezoelectric coefficients and piezopotentials of the polarized PVDF solid electrolytes. The higher piezopotential from the solid electrolyte is expected to enable the migration of more Li^+^ from the cathode to anode, thereby promoting energy conversion/storage efficiency.

### 3.3 Voltage‐Responsive Electrolytes for Overcharging‐Protection EES Devices

Overcharge is an abnormal and often‐seen charging state at which the electricity flow is forced into a fully charged EES device.^[^ ^66^ ^]^ Usually, EES devices get overcharged when the voltage is incorrectly detected by the charging control system or when the charger breaks down. Overcharge may cause irreversible electrode structure degradation, electrolyte decomposition, as well as gas production. The excessive heat and gas generated from these side reactions would induce rapid device performance failure and increase the risk of fire and explosion. To this end, voltage‐responsive electrolytes have been used to prevent the overcharge of EES devices (particularly for rechargeable batteries). Until now, three types of voltage‐responsive electrolytes have been developed, and their working principles rely on redox shuttle additives, current leakage additives, and ion diffusion inhibiting additives, respectively.

#### 3.3.1 Voltage‐Responsive Electrolytes based on Redox Shuttle Additives

**Figure 6:**
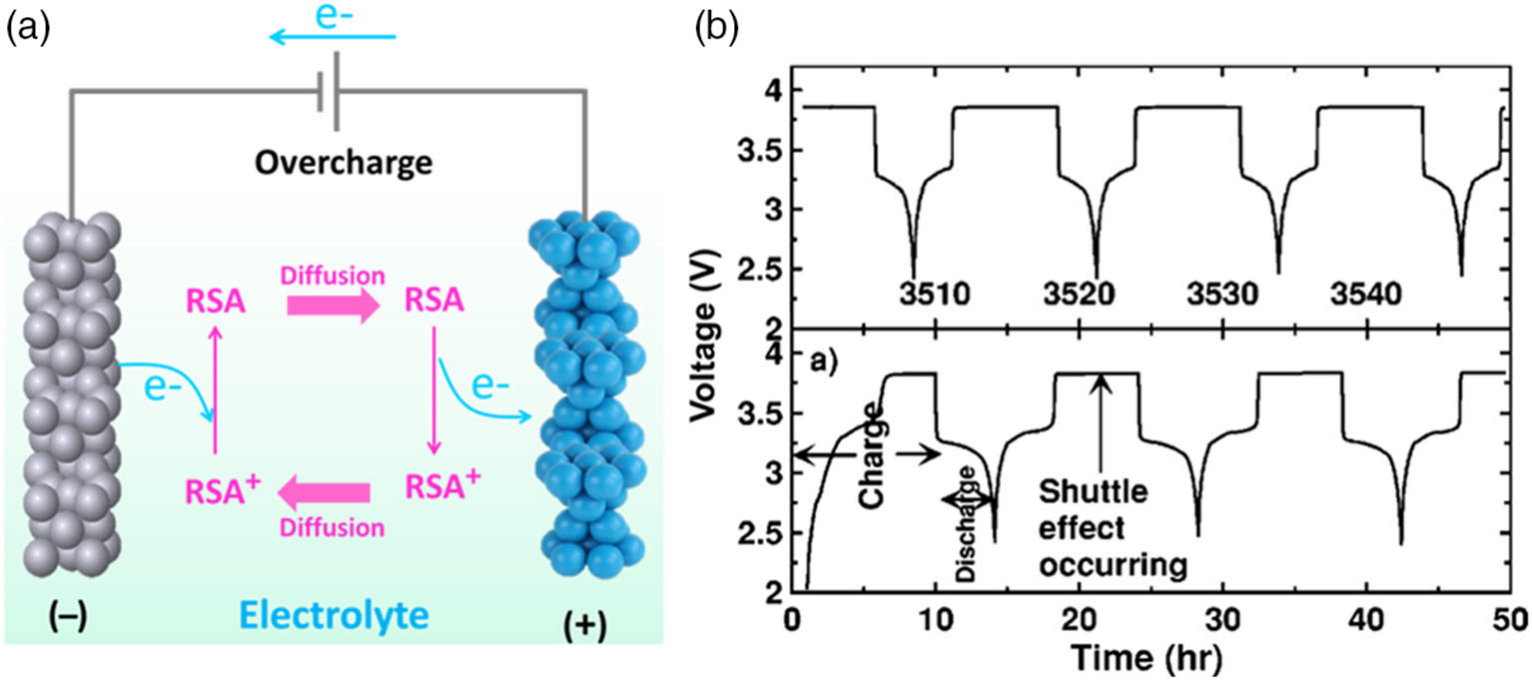
a) Schematic illustration showing the working principle of RSAs in voltage‐responsive electrolytes. b) The charge/discharge curves of the graphite||LiFePO_4_ battery fabricated with the voltage‐responsive electrolyte based on the RSAs. b) Reproduced with permission^[^ ^74^ ^]^ Copyright 2005, The Electrochemical Society.

Overcharge protection is particularly important for module‐ and pack‐level applications of Li‐ion batteries. Monitoring the state of charge (SoC) of all cells in the pack in real time remains highly challenging. In practical applications, estimation of SoC relies on the use of voltage sensors outside the battery, which cannot realize the accurate detection of the internal SoC in batteries. It is common to see that some batteries in a pack have lower capacities than others. When the pack is charged, batteries with lower capacity will first reach the fully charged state, while other batteries in the pack are not fully charged. In this regard, the charger will continue to charge the pack as scheduled, and the fully charged batteries would be overcharged. To address this obstacle, redox shuttle additives (RSAs) were introduced into electrolytes to control the charging voltages of Li‐ion batteries.^[^ ^67^, ^68^ ^]^ The RSAs are usually organic molecules or polymers, which have slightly higher oxidized and reduced potentials than the maximum operating potentials of the cathodes. Under normal operation, the RSAs are inactive. Once the Li‐ion batteries are overcharged, RSAs would be preferentially oxidized on the cathodes to consume the extra electricity and protect the batteries from overcharge. The oxidized RSAs would next travel to the anode side across the electrolyte and be reduced. The reduced RSAs would then diffuse back to the cathode (**Figure** **6**a) and be oxidized again.^[^ ^69^, ^70^ ^]^

In the early research, RSAs utilized for overcharge‐protection Li‐ion batteries include LiI and ferrocene derivatives.^[^ ^71^, ^72^ ^]^ Due to the low redox potentials, these inorganic additives only provided the overvoltage‐protection function at a voltage range of 3–3.5 V versus Li/Li^+^. This low voltage range cannot match with the commercial Li‐ion batteries, which generally have a large cutoff voltage of 4.2 V. Later, RSAs with high redox potentials of more than 4 V were explored.^[^ ^67^, ^68^, ^73^, ^74^ ^]^ In 2005, Dahn and coworkers^[^ ^73^ ^]^ evaluated a series of organic molecules as RSAs for the overcharge protection of commercial Li‐ion batteries. Among all the tested molecules, 2,5‐di‐*tert*butyl‐1,4‐dimethoxybenzene displayed the best overcharge protection ability. Figure 6b shows the overcharge‐protection behavior of the constructed graphite||LiFePO_4_ battery. The redox potential of 2,5‐di*tert*butyl‐1,4‐dimethoxybenzene is close to 4 V versus Li/Li^+^. The charging voltage of the graphite||LiFePO_4_ battery was stabilized at about 4 V versus Li/Li^+^ during the charging process, which was effectively controlled in an acceptable region. Moreover, the overcharge‐protection function of the fabricated battery was able to last continuously for over 300 cycles.^[^ ^74^ ^]^ Apart from the aromatic organic compounds, some nonaromatic compounds were also proposed as RSAs, such as tetramethylpiperidinooxy (TEMPO) and organometallic compounds.^[^ ^75^, ^76^ ^]^ All these RSAs can protect against the overcharge of Li‐ion batteries in a similar manner to the aromatic molecules.

#### 3.3.2 Voltage‐Responsive Electrolytes based on Current Leakage Additives

Another type of additives for voltage‐responsive electrolytes protects EES device by inducing the current leakage at the overcharge conditions. Voltage‐responsive electrolytes based on current leakage additives can undergo the conversion from the ionic conducting state to the electrical conducting state when EES devices are overcharged.^[^ ^77^ ^]^ The electrical conducting state of the electrolyte leads to the weak short circuit and current leakage, protecting the EES devices from further overcharge. Such functional electrolytes rely on the use of the p‐type electroactive poly(3‐butylthiophene) (P3BT) additive, which can rapidly switch between the ionic conducting state and electrical conducting state due to the anion doping/dedoping reaction.^[^ ^78^, ^79^ ^]^ The electrical conductivity of P3BT at the ionic conducting state was very low (only 10^−6^ mS cm^−1^).^[^ ^80^ ^]^ After anion doping at high potentials, the electrical conductivity of P3BT reached up to 100 mS cm^−1^. P3BT was subsequently integrated into the gel–polymer electrolyte for the Li||LiCoO_2_ battery.^[^ ^77^ ^]^ The insulating feature enabled P3BT to act like a normal polymer matrix at room temperature, allowing the obtained gel–polymer electrolyte with favorable Li^+^ transport capability. When the Li||LiCoO_2_ battery was overcharged to 4.2 V, P3BT was oxidized with the anion (PF_6_^−^) doping reaction, leading to the increase in the electrical conductivity of the gel–polymer electrolyte. Consequently, the weak internal short circuit in the Li||LiCoO_2_ battery was induced by the electrical conducting P3BT, which triggered the current leakage to consume the extra electricity and protect the Li||LiCoO_2_ battery from further voltage runaway.^[^ ^77^, ^78^, ^79^ ^]^

Apart from P3BT, other conducting polymers, such as poly (4‐methoxytriphenylamine),^[^ ^81^ ^]^ polytriphenylamine,^[^ ^82^, ^83^ ^]^ and poly(3‐decyl thiophene),^[^ ^84^ ^]^ were also demonstrated as current leakage additives for the voltage‐responsive electrolytes of Li‐ion batteries. Their cutoff voltages for overcharge protection vary according to their doping/dedoping potentials. Similar to the RSAs,^[^ ^73^, ^74^, ^75^, ^76^ ^]^ the redox potentials of these p‐type electroactive polymers should be slightly higher than the normal working potentials of battery cathodes.^[^ ^77^, ^78^, ^79^, ^80^, ^81^, ^82^, ^83^, ^84^ ^]^ However, it should be pointed out that triggering the weak short circuit at the overcharge state would raise the risk of thermal runaway, which could induce more serious safety incidents of EES devices.

#### 3.3.3 Voltage‐Responsive Electrolytes based on the Ion Diffusion Inhibiting Additives

The third type of voltage‐responsive electrolytes protects EES devices from overcharge by using electrolyte additives to inhibit ion transport in the devices. Classic examples are the functional electrolytes based on the aromatic organic additives (e.g., biphenyl, xylene, 3‐chlorothiophene, and furan) for the overcharge protection of Li‐ion batteries.^[^ ^85^, ^86^, ^87^ ^]^ These aromatic organic additives can be electrochemically polymerized on the electrode surface at the high charging voltages of Li‐ion batteries. The formed polymer layers would block the transport of Li^+^ at the electrode/electrolyte interface, thereby switching off the batteries at the high charging voltages. For example, the electrochemical polymerization of the xylene electrolyte additive was demonstrated on the overcharged LiCoO_2_ cathodes at the high potential of >4.5 V versus Li/Li^+^, generating an isolating polymer film on the cathode surface and blocking the Li^+^ transport at the cathode/electrolyte interface.^[^ ^85^ ^]^ Nevertheless, the electrochemical polymerization reactions of additives are irreversible, which may lead to switch off the battery permanently. Moreover, the polymerization process could spontaneously release a large amount of heat and gas, leading to the increase in internal temperature and pressure.

**Figure 7:**
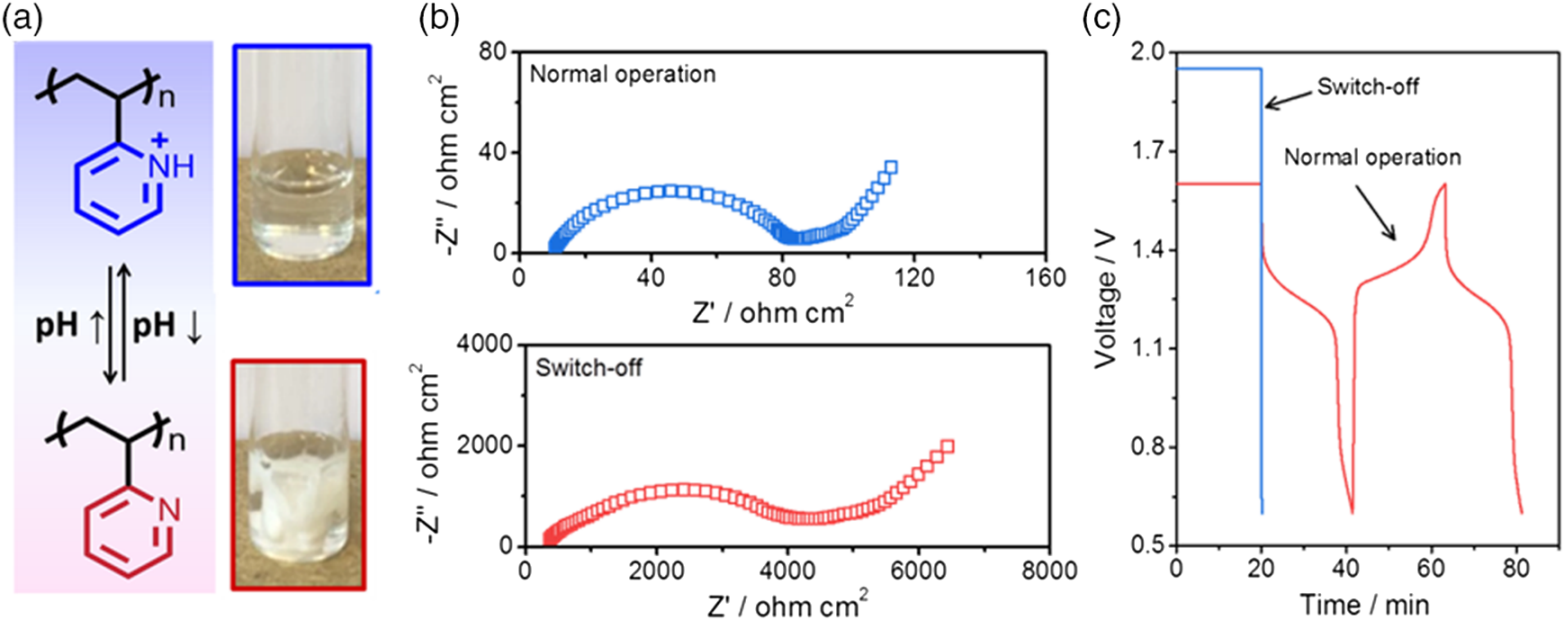
Voltage‐responsive electrolyte based on the ion diffusion inhibiting additive for Zn‐ion aqueous batteries. a) Schematic showing the structure transition of P2VP from the hydrophilic state to the hydrophobic state. b) Electrochemical impedance spectra and c) voltage–time curves of the assembled Zn‐ion aqueous batteries after constant‐voltage charge at 1.6 and 1.95 V. a–c)Reproduced with permission.^[^ ^88^ ^]^ Copyright 2020, Wiley‐VCH.

In addition to Li‐ion batteries, an overcharge self‐protection strategy was also applied for aqueous batteries.^[^ ^88^ ^]^ Overcharge in aqueous batteries usually leads to irreversible decomposition of water, like O_2_ evolution at the cathode and H_2_ evolution at the anode. Most recently, our studies on Zn‐ion aqueous batteries with the I_2_/C cathode showed that the overcharge induced fast battery failure with severe performance deterioration, which can be assigned to the irreversible formation of by‐product ZnO at the anode and soluble Zn(IO_3_)_2_ at the cathode. Moreover, the gas evolution at the overcharge state caused the internal pressure rise and swelling/cracking of the battery pack. To prevent the battery failure at the overcharge state, several voltage‐responsive electrolytes were prepared based on ZnSO_4_ solution and a series of polymers with tertiary amine or pyridinic groups. It was demonstrated that the electrolyte based on poly(2‐vinylpyridine) (P2VP) showed the best overcharge‐protection capability for Zn‐ion aqueous batteries with rapid response time (less than 30 s).^[^ ^88^ ^]^ The H_2_ evolution side reaction at the overcharge state induced the increase in the local pH near the Zn anode surface, which led to the deprotonation of quaternary pyridinic groups in P2VP and the subsequent phase separation of P2VP‐based electrolyte from the hydrophilic state to the hydrophobic state (**Figure** **7**a). The precipitated P2VP solid on the Zn metal surface further blocked Zn^2+^ transportation at the electrolyte/electrode interface and increased the charge transfer resistance of the batteries by two orders of magnitude (Figure 7b). As a result, the assembled Zn‐ion aqueous batteries under the overcharge conditions were rapidly turned off (Figure 7c), preventing the electrodes from irreversible structural damage.^[^ ^88^ ^]^ Importantly, such voltage‐responsive electrolytes showed the repeatable overcharge‐protection capability for several times in light of the reversible hydrophilic‐to‐hydrophobic phase separation of the P2VP‐based electrolyte. When the electrolyte pH was re‐adjusted to the initial value, the precipitated P2VP was redissolved into the electrolyte, and the electrode/electrolyte interface became ionic conductive again.

### 3.4 Other Responsive Electrolytes for Smart EES Devices

In addition, magnetism‐ and sunlight‐responsive electrolytes were developed to prevent the electrolyte leakage and reduce the charging overpotential of EES devices.^[^ ^89^, ^90^ ^]^ The magnetism‐responsive electrolyte was prepared by dispersing silica‐coated magnetic Fe_3_O_4_ nanoparticles into ionic liquid 1‐ethyl‐3‐methylimidazolium bis(trifluoromethylsulfonyl)imide (EMITFSI).^[^ ^89^ ^]^ The obtained silica/Fe_3_O_4_/EMITFSI electrolyte is one type of magneto‐rheological fluids, which exhibited rapid and reversible phase change from liquid to solid under an external magnetic field. The liquid–solid phase transition was associated with the chain‐like alignment of silica/Fe_3_O_4_ nanoparticles caused by the dipolar interaction.^[^ ^91^, ^92^ ^]^ Typically, the viscosity of magnet‐responsive silica/Fe_3_O_4_/EMITFSI electrolyte was low (≈200 Pa s) without the magnetic field and increased substantially to 20 000 Pa s when the magnetic field was imposed. It is worth noting that the silica/Fe_3_O_4_ nanoparticles formed a stable chain‐like structure in the liquid EMITFSI along the direction of the applied magnetic field. The mobility of electrolyte ions was not affected by the alignment of the magnetic silica/Fe_3_O_4_ nanoparticles. Consequently, the magnetically induced phase transition had almost no influence on the ionic conductivity of the silica/Fe_3_O_4_/EMITFSI electrolyte.^[^ ^89^ ^]^ Thereby, this silica/Fe_3_O_4_/EMITFSI electrolyte would be inapplicable for the magnetism‐controlled switch on/off of EES devices. Instead, it could be potentially used to prevent electrolyte leakage when the pack of EES devices is broken. In addition, separate solid metallic magnets are widely utilized as fillers into matrices to fabricate microwave absorption materials. Sun et al. pioneered the field of magnetized CNT microwave absorber with tunable microwave absorption frequencies.^[^ ^93^ ^]^ Although it has not yet been reported to electrolytes and electrode materials, the integration of microwave absorption functions into EES devices has potentially wide application for the high‐power electronic instruments in the fields of electronic safety and defense stealth technology.

**Figure 8:**
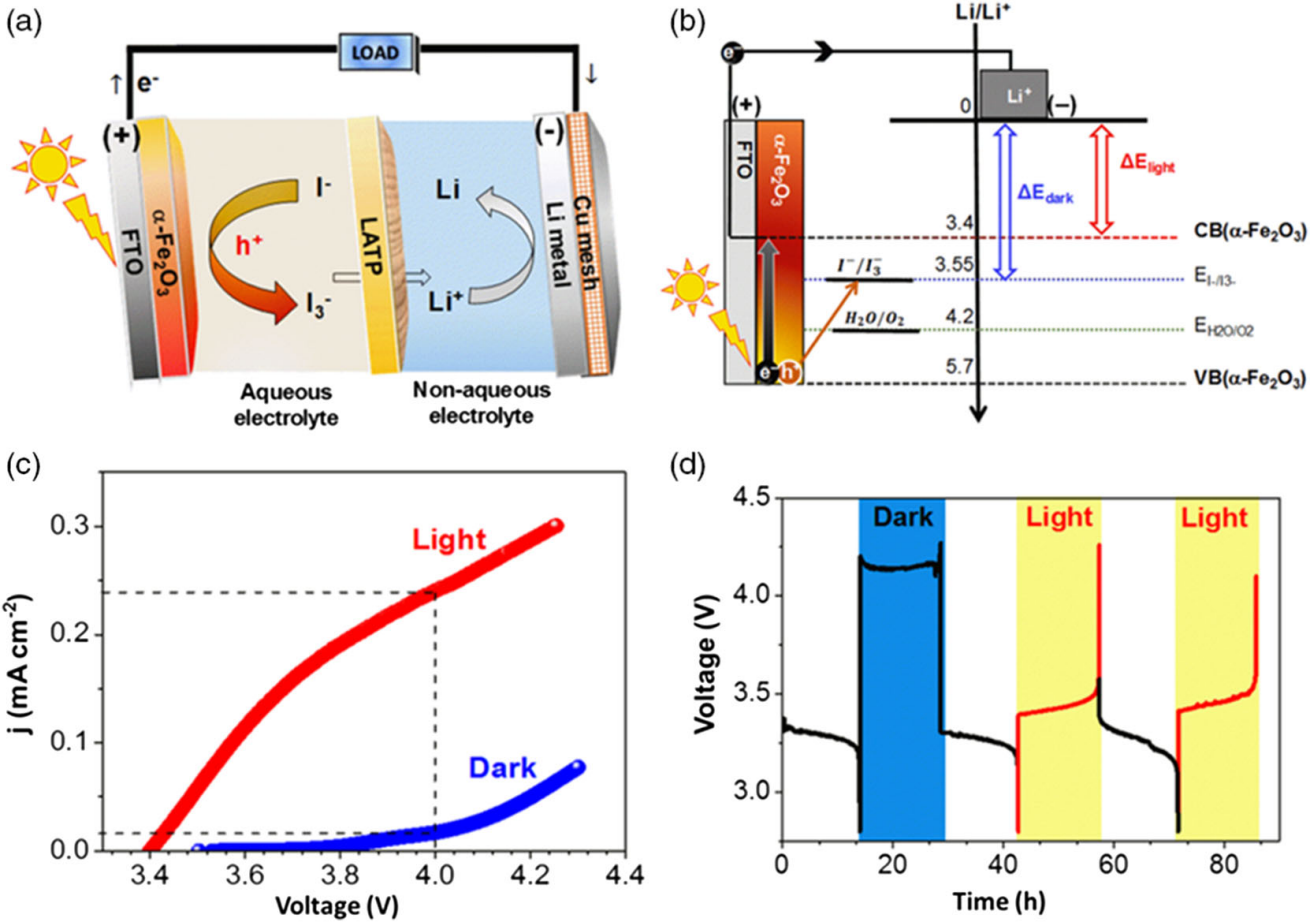
Sunlight‐responsive electrolyte for Li‐I_2_ batteries. a) Schematic illustrating the structure of the assembled Li–I_2_ batteries. b) The working principle of the light response of the electrolyte and hematite electrode in the assembled Li–I_2_ batteries. c) The anodic linear sweep voltammetry and d) charge/discharge profiles of the assembled Li–I_2_ batteries under solar and dark conditions. All discharge processes were conducted at the dark conditions. a–d) Reproduced with permission.^[^ ^90^ ^]^ Copyright 2016, American Chemical Society.

Integration of EES devices with sunlight‐responsive behaviors is a feasible strategy for the utilization of clean and renewable solar energy.^[^ ^94^, ^95^, ^96^ ^]^ Currently, the reported sunlight‐responsive electrolytes generally contain aqueous I^−^/I_3_^−^ redox couples. The liquid I^−^/I_3_^−^ redox couple can serve as not only an important component of dye‐sensitized solar cell electrolytes but also as the catholyte in metal–I_2_ batteries. With the help of photoelectrodes, the electrolyte containing I^−^/I_3_^−^ redox couple can respond to sunlight. In 2016, Byon and coworkers^[^ ^90^ ^]^ demonstrated a sunlight‐responsive Li–I_2_ battery (**Figure** **8**a) using the hematite (α‐Fe_2_O_3_) photoelectrode and LiI/LiI_3_ catholyte. Upon illumination, the photon (*hv*) absorbed by the hematite electrode generated electrons (e^−^) and holes (h^+^) as charge carriers. The majority e^−^ carriers at the quasi‐Fermi level of hematite jumped to the conduction band (CB) edge under the sunlight, whereas the quasi‐Fermi level for the minority h^+^ carriers aligned with the potential of the I^−^/I_3_^−^ redox reaction (Figure 8b). When e^−^ was excited to the CB edge of the hematite photoelectrode, the accumulated free h^+^ at the quasi‐Fermi level of hematite diffused through the interface to promote the electrochemical I^−^ oxidation to I_3_^−^. This photoassisted process shifted the onset oxidation potential of the I^−^/I_3_^−^ redox couple to 3.4 V versus Li^+^/Li, which was apparently lower than that (3.85 V vs Li/Li^+^) of the I^−^/I_3_^−^ redox couple in the absence of light assistance (Figure 8c). Furthermore, the charging overpotential decrease in the light‐responsive Li–I_2_ battery was evidenced by the charge/discharge profiles (Figure 8d). Under the dark condition, the charging voltage of the Li–I_2_ battery was 4.1 V versus Li/Li^+^ at a current density of 0.075 mA cm^−2^. Upon illumination, the charging voltage of the Li–I_2_ battery was markedly reduced to 3.43 V versus Li/Li^+^. The photogenerated charge carriers reduced the charging overpotential by 0.66 V, enabling remarkably improved voltage and energy efficiencies.

## 4 Smart Electrochromic Electrolytes for EES

It is common to visualize color change of materials along with their chemical reduction/oxidation processes. Electrochromic materials refer to materials whose color changes are induced by passing a current or applying a potential.^[^ ^97^, ^98^ ^]^ In light of the reversible redox reactions, electrochromic materials can be directly utilized as active electrode materials for pseudocapacitors or rechargeable batteries.^[^ ^99^, ^100^, ^101^ ^]^ Integrating electrochromic behavior into EES devices is highly attractive to straightforwardly monitor the charging/discharging states in a visible and predictable manner.

**Figure 9:**
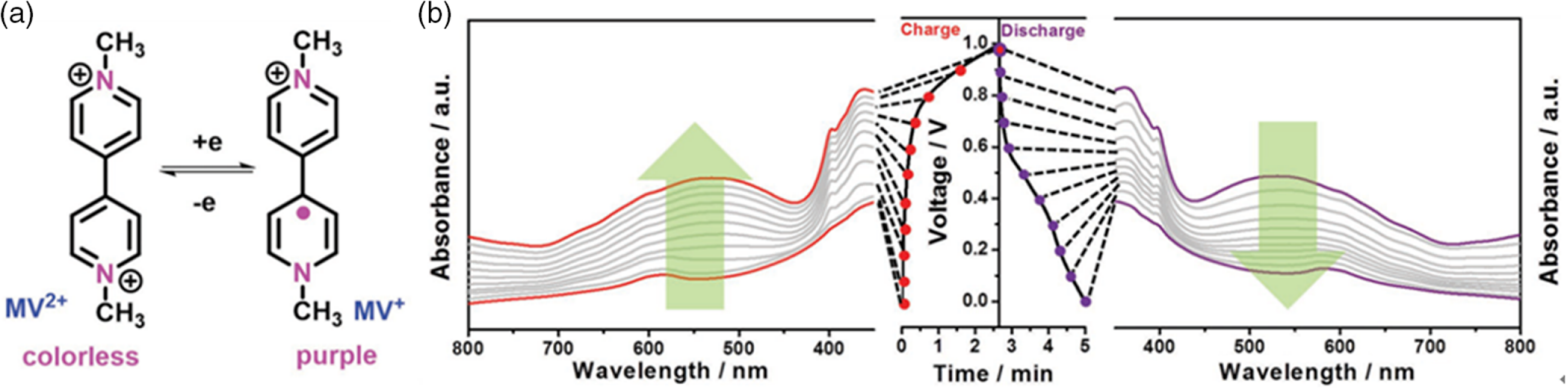
Electrochromic electrolyte based on MV for microsupercapacitors. a) Electrochromic mechanism of the electrolyte based on MV. b) The UV–vis absorption spectra of the assembled microsupercapacitor with the MV‐based electrochromic electrolyte in the voltage range of 0–1 V. a,b) Reproduced with permission.^[^ ^102^ ^]^ Copyright 2017, Wiley‐VCH.

Commonly, electrochromic EES devices are constructed by depositing electrochromic electrode materials on transparent current collectors. In a different way, our group reported a microsupercapacitor based on an electrochromic electrolyte.^[^ ^102^ ^]^ Specifically, methyl viologen (MV) mixed with the PVA/LiCl hydrogel was used as the electrochromic electrolyte for the symmetric microsupercapacitor assembled with two graphene/V_2_O_5_ electrodes. The electrochromic function of the MV/PVA/LiCl electrolyte originated from the reversible redox reaction between the colorless viologen and the purple radical viologen derivative (**Figure** **9**a) in the voltage window of 0–1 V. The fabricated micro‐supercapacitor delivered a high volumetric energy density of 20 mWh cm^−3^ at a power density of 235 W cm^−3^. During the charging process, the color of the used electrolytes in microsupercapacitors gradually became deeper, and the deepest purple color was observed when the voltage reached 1 V. During the discharging process, the color of the electrolyte gradually fades to colorless at 0 V. Accordingly, the electrochromic phenomenon of the used electrolyte was evidenced by the in situ UV–vis absorbance tests, as shown in Figure 9b. The introduced electrochromism in the electrolyte can realize the real‐time display of the charging/discharging status of EES devices, enhancing the human–device interaction experience.^[^ ^102^ ^]^

Apart from electrochromic supercapacitors, electrochromic batteries are also fascinating smart devices. In 2014, a new electrochromic battery with the self‐powered function was designed with the aluminum (Al) anode, Prussian blue (PB) cathode, and KCl aqueous electrolyte.^[^ ^103^ ^]^ During the discharge process, the PB cathode was bleached to Prussian white (PW, colorless). More interestingly, the spontaneous oxidation of PW to PB by the O_2_ in the air triggered the Al||PB battery to be self‐charged. However, the electrochromic process of the Al||PB battery took a very long time (about 12 h) due to the low charge transfer rate of the spontaneous oxidation reaction of PW.

Recently, a new aqueous electrolyte containing a trace amount of H_2_O_2_ additive was developed for the electrochromic Al||WO_3_ battery with the self‐powered function.^[^ ^104^ ^]^ The addition of H_2_O_2_ into the electrolyte greatly accelerated the spontaneous oxidation reaction of the reduced cathode (Al_*x*_WO_3_) in the Al||WO_3_ battery due to the transition of the energy level from O^2^/O^2−^ to H_2_O_2_/H_2_O. With the assistance of the H_2_O_2_ additive, the colorless‐to‐blue transition process in the assembled electrochromic battery was achieved in only 8 s. In addition, the H_2_O_2_ additive significantly improved the discharge capacity of the electrochromic battery to 429 mAh g^−1^, which is beyond the theoretical discharge capacity (353 mAh g^−1^). It can be explained by the extra electron transfer caused by the formation of peroxo acid on the cathode. The strategy of using electrolyte additives brings new insights into the design of electrochromic batteries with high capacities.

## 5 Smart Self‐Healing Electrolytes for EES

EES devices usually suffer from internal or external damage when they are practically applied in flexible and stretchable electronics. The complex and deformable environments (e.g., bending, stretching, and twisting) may lead to damages and cracks inside the EES devices, which are hard to be detected and repaired. Toward this end, EES devices are equipped with self‐healing function inspired by the natural self‐healing ability of tissue and skin.^[^ ^15^ ^]^ Endowing EES devices with the self‐healing function can automatically repair the internal or external cracks and breaks in EES devices, significantly enhancing the durability and lifespan of EES devices. Early reports on self‐healing EES devices mainly focused on the self‐recovery of the electrode or device integrity.^[^ ^15^, ^105^, ^106^, ^107^ ^]^ However, the lack of self‐repair capability in electrolytes limits the healing efficiencies of EES devices after deformation. To achieve better self‐healing efficiency of EES devices, various electrolytes with intrinsically self‐healing function have been developed over the last decade. All reported self‐healing electrolytes are semisolid, quasisolid, or all‐solid electrolytes. Currently, the used self‐healing electrolytes in EES devices can effectively reconstruct the broken interfaces via interactions like hydrogen bonds, dynamic covalent bonds, and electrostatic interactions. **Table** **2** shows the basic characteristics of the recently reported self‐healing electrolytes and the electrochemical properties of the assembled EES devices.

**Table 2:** Comparisons of various EES devices with intrinsically self‐healing electrolytes

Electrolyte	EES device	Self‐healing mechanism	Self‐healing time	Ionic conductivity retention	Capacity retention	Ref.
PAA/VSNPs/H_3_PO_4_	PPy/CNT‐based symmetric supercapacitors	Hydrogen bonds	–	>90% (4th)	≈100% (20th)	108
PAA/MGO/H_2_SO_4_	PPy/CNT‐based symmetric supercapacitors	Hydrogen bonds	≈30 min	>85% (5th)	>60% (5th)	109
PVA/Zn(CF_3_SO_3_)_2_	Zn ||PANI/CNT Zn‐ion batteries	Hydrogen bonds	≈10 min	>60% (10th)	≈100% (3th)	110
PVA‐g‐PAA/borax/KCl	AC‐based symmetric supercapacitors	Borate ester bonds and hydrogen bonds	≈10 min	–	≈100% (10th)	115
SA‐g‐DA/borax/KCl	AC‐based symmetric supercapacitors	Borate ester bonds and hydrogen bonds	≈15 min	≈100% (5th)	≈95% (10th)	116
SA‐g‐DA/borax/Li_2_SO_4_	LiV_3_O_8_||LiMn_2_O_4_ aqueous Li‐ion batteries	Borate ester bonds and hydrogen bonds	≈15 min	–	≈60% (10th)	117
PANa/FeCl_3_/KOH/Zn(OH)_4_^2+^	Zn||NiCoO alkaline batteries	Electrostatic interactions	–	>95% (5th)	≈87% (4th)	119

### 5.1 Self‐Healing Electrolytes based on Hydrogen Bonds

**Figure 10:**
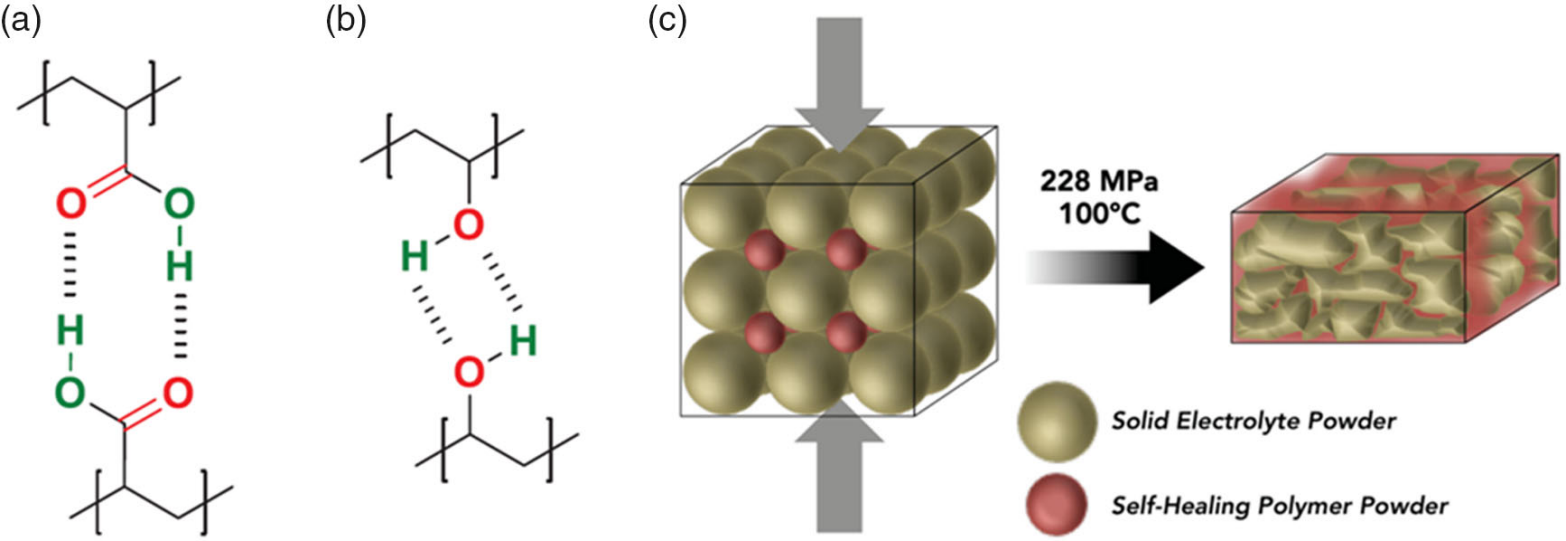
Hydrogen bond‐based self‐healing electrolytes. Molecular structures of self‐healing a) PAA and b) PVA with hydrogen bonds. Reproduced with permission.^[^ ^15^ ^]^ Copyright 2020, Wiley‐VCH. c) Schematic of a scalable method for forming the solid‐state electrolyte in self‐healing polymer matrix. Reproduced with permission.^[^ ^111^ ^]^ Copyright 2015, Wiley‐VCH.

Self‐healing electrolytes based on hydrogen bonds are the most common electrolytes for EES devices, which consist of polymers equipped with functional groups like carboxyl, amide, urea pyrimidine, and hydroxyl. In 2015, Zhi and coworkers^[^ ^108^ ^]^ developed a self‐healing electrolyte composed of polyacrylic acid (PAA) and a small amount of vinyl hybrid silica nanoparticles (VSNPs). Importantly, the PAA polymer chains provided sufficient hydrogen bonding interactions (**Figure** **10**a). The physical crosslink by the reversible hydrogen bonds and the covalent crosslink by VSNPs ensured the superior self‐healing property.^[^ ^15^ ^]^ The assembled symmetric supercapacitor based on the PAA/VSNPs/H_3_PO_4_ electrolyte achieved a high healing efficiency of 100% during 20 breaking/healing cycles. Similarly, the methacrylated graphene oxide (MGO)‐crosslinked PAA electrolyte was used to construct self‐healing supercapacitors.^[^ ^109^ ^]^ MGO was demonstrated to promote the formation of hydrogen‐bonded networks among PAA chains and act as the crosslinking mediator to improve the mechanical property of the electrolyte.^[^ ^15^ ^]^ The self‐healing PAA–MGO electrolyte showed an ionic conductivity of up to 71.6 mS cm^−1^,^[^ ^109^ ^]^ which was much more conductive than the PAA–VSNPs electrolyte (≈7.5 mS cm^−1^).^[^ ^108^ ^]^

Apart from supercapacitors, self‐healing electrolytes based on hydrogen bonds have also been widely employed for metal‐ion batteries. In 2019, Chen and coworkers^[^ ^110^ ^]^ reported the self‐healing PVA/Zn(CF_3_SO_3_)_2_ hydrogel electrolyte for Zn‐ion batteries. The PVA chain segments are equipped with rich hydroxyl side groups to form hydrogen bonds, which further enable the PVA‐based hydrogel with outstanding self‐healing capability (Figure 10b). The developed PVA/Zn(CF_3_SO_3_)_2_ hydrogel electrolyte showed a wide electrochemical window with the oxygen evolution potential suppressed up to 2.25 V versus Zn/Zn^2+^. A stable Zn anode with low overpotential over 800 cycles was realized using PVA/Zn(CF_3_SO_3_)_2_ hydrogel electrolyte. The fabricated Zn‐ion battery based on polyaniline cathode exhibited a specific capacity of 94 mAh g^−1^ at a high current density up to 3 A g^−1^. More importantly, the resultant Zn‐ion batteries could completely restore the electrochemical performance after three repeating cutting/healing processes.^[^ ^110^ ^]^

In addition, self‐healing nonaqueous electrolytes were developed based on polyimine and supramolecular rubber.^[^ ^111^, ^112^ ^]^ For example, Lee et al. filled polyimine matrix into the void of the nonaqueous Li_2_S–P_2_S_5_ solid‐state electrolyte using a simple hot isostatic press technology (Figure 9c). Due to the self‐healing property of the imine bonds of polyimine, a uniformly mixed organic/inorganic matrix was formed and worked as an ultrathin solid‐state electrolyte. With the obtained Li_2_S–P_2_S_5_/polyimine membrane as the electrolyte, the fabricated all‐solid‐state Li‐ion battery with the FeS_2_ cathode exhibited excellent stability with capacity retention of 74% over 200 cycles,^[^ ^111^ ^]^ significantly outperforming the FeS_2_‐based all‐solid‐state battery with the pure Li_2_S–P_2_S_5_ solid electrolyte (less than 30 cycles).^[^ ^113^ ^]^ The outstanding stability of the all‐solid‐state Li||FeS_2_ battery was assigned to the self‐healing polyimine, which could quickly restore the structural integrity and electrochemical/mechanical properties of the solid‐state Li_2_S–P_2_S_5_ electrolyte during the Li^+^ transport. Likely, supramolecular rubber was also explored as an alternative self‐healing polymer matrix for inorganic solid‐state electrolytes due to its superior self‐healing function and the excellent volumetric accommodation to negate the strain accumulation.^[^ ^112^, ^114^ ^]^ A smooth surface of Li metal anode was still retained in the self‐healing supramolecular rubber/Ga‐doped Li_7_La_3_Zr_2_O_12_ electrolyte for 1500 stripping/plating cycles at an ultrahigh current density of 20 mA cm^−2^.^[^ ^112^ ^]^ The urea (–—NH—CO—NH—) and acylamide (—NH—CO—) groups of supramolecular rubber could form multiple hydrogen bonds between the N—H donors and C=O acceptors, subsequently providing outstanding self‐healing property and avoiding the formation of cracks in Li anode.

### 5.2 Self‐Healing Electrolytes based on Covalent Bonds

**Figure 11:**
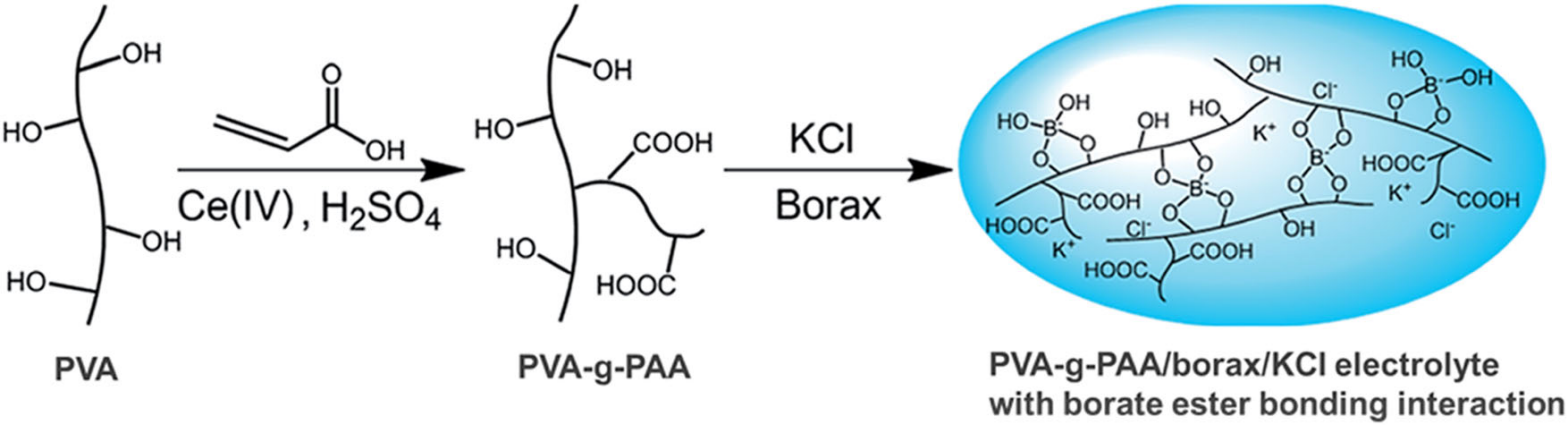
Self‐healing PVA‐g‐PAA/borax/KCl hydrogel electrolyte based on borate ester bonds. Reproduced with permission.^[^ ^115^ ^]^ Copyright 2016, The Royal Society of Chemistry.

The self‐healing processes based on covalent bonds involve the reversible formation of inherent dynamic covalent bonds. Currently, borate ester chemistry has been introduced into hydrogen bond‐based self‐healing electrolytes to further enhance the self‐healing ability.^[^ ^115^, ^116^, ^117^ ^]^ Borate ester bonds are reversible dynamic covalent bonds formed by the condensation reaction of boric acid with 1,2‐ or 1,3‐diols under alkaline conditions. In 2016, Pan and coworkers^[^ ^115^ ^]^ reported a self‐healing PVA‐g‐PAA/borax/KCl hydrogel electrolyte relying on dynamic diol‐borate ester bonds. Specifically, PAA was first grafted on PVA to form PVA‐g‐PAA copolymer. The grafting of PAA alleviated the coagulation of PVA chains caused by the salts. The PVA‐g‐PAA copolymer was then converted to transparent PVA‐g‐PAA/borax/KCl hydrogel electrolyte in the presence of borax and KCl (**Figure** **11**). To evaluate the self‐healing capability, the PVA‐g‐PAA/borax/KCl hydrogel electrolyte was cut into three parts. Impressively, these three parts were able to be self‐repaired quickly once they are contacted with each other. The fabricated supercapacitors with the PVA‐g‐PAA/borax/KCl electrolyte showed no significant capacitance deterioration even after ten cutting–healing cycles.^[^ ^115^ ^]^

The self‐healing hydrogel electrolyte based on borate ester bonds was also utilized to assemble aqueous Li‐ion batteries.^[^ ^117^ ^]^ In detail, the hydrogel electrolyte was prepared by crosslinking dopamine‐grafted sodium alginate (SA‐g‐DA) via dynamic catechol‐borate ester bonds in the presence of borax and Li_2_SO_4_. When the electrolyte was mechanically damaged, catechol‐borate ester bonds could be immediately formed at the contact interface, effectively reconstructing the broken interface and restoring the ion transport. A fully self‐healable aqueous Li‐ion battery was demonstrated using this hydrogel electrolyte together with the self‐healing PVA‐based LiV_3_O_8_ anode and LiMn_2_O_4_ cathode. In the cutting/healing tests, the damaged aqueous Li‐ion battery was able to recover 98.7% of its original tensile strength after 20 min of self‐healing.^[^ ^117^ ^]^

### 5.3 Self‐Healing Electrolytes based on Electrostatic Interactions

Electrostatic interactions between the negatively charged polymer chains and metal cations can also be utilized to construct self‐healing electrolytes. The polymer matrix in electrolytes can break and reform reversibly because the electrostatic interactions serve as the crosslinkers of the polymer matrix.^[^ ^118^, ^119^ ^]^ A classic example is the ferric‐ion (Fe^3+^)‐crosslinked sodium polyacrylate (PANa) hydrogel electrolyte for aqueous alkaline batteries.^[^ ^119^ ^]^ PANa served as the hydrogel matrix for free‐moving OH^−^ with high ionic conductivity. Meanwhile, Fe^3+^ served as the crosslinker to reinforce the self‐healing property of the PANa hydrogel. The electrostatic interaction between Fe^3+^ and the acrylate groups of PANa could be broken and reformed reversibly. Importantly, the as‐fabricated Zn‐alkaline battery with the Zn/carbon cloth anode and the NiCoO/carbon cathode maintained 87% of its initial capacity after four cutting–healing cycles.^[^ ^119^ ^]^ The slight deterioration of the capacity was assigned to the gradually increased interfacial resistance between the electrodes and the hydrogel electrolyte.

In addition, the electrostatic interactions based on the ion‐dipole mechanism were also used for constructing self‐healing electrolytes. Ion–dipole interactions are electrostatic interactions between charged ions and neutral polar polymers. In 2018, Jin and coworkers^[^ ^120^ ^]^ reported an IL‐immobilized polymer electrolyte with strong ion–dipole interaction for the self‐healing function. The ion–dipole interaction was formed between the positively charged imidazolium cations of IL and the strongly electronegative fluorine atoms of PVDF‐HFP. The desirable ion–dipole interaction endowed the polymer electrolyte with remarkably improved mechanical strength and self‐healing capability. This self‐healing polymer electrolyte with 40 wt% of IL had a high ionic conductivity of close to 0.88 mS cm^−1^ at room temperature, which reached the level of conventional gel‐polymer electrolytes or liquid electrolytes.^[^ ^121^, ^122^, ^123^ ^]^ The prepared self‐healing IL/PVDF polymer electrolyte was demonstrated to effectively suppress the lithium dendrite growth. During the lithium stripping/plating process, the electrolyte could withstand the mechanical deformation caused by the volume change and avoid the formations of cracks on the surface of Li metal anode. At a high areal capacity of 1 mAh cm^−2^ per cycle, the fabricated Li||Li cell for the evaluation of the Li anode exhibited a stable voltage plateau for 1000 h without short circuiting.^[^ ^120^ ^]^

## 6 Conclusion

In conclusion, stimulus‐responsive electrolytes with sensitive response to temperature, mechanical force, voltage, light, magnetism, and structural integrity, electrochromic electrolytes, and self‐healing electrolytes have been successfully demonstrated in EES devices, showing diverse smart functions. Apart from the basic function of storing electrical energy, EES devices integrated with smart functions can address many challenging problems and provide new opportunities in practical applications. In detail, i) temperature‐ and voltage‐responsive electrolytes were used to prevent the overheating and overcharging hazards of EES devices. ii) Mechanical force‐responsive electrolytes enabled EES devices with self‐powered capability. iii) Sunlight‐responsive electrolytes were beneficial for improving the electrochemical properties of EES devices by decreasing the charging overpotentials. iv) Magnetism‐responsive electrolytes were used to protect the liquid electrolyte in EES devices from leakage. v) Electrochromic electrolytes provided the straightforward visualization for the energy storage state of EES devices without the aid of extra techniques. vi) Self‐healing electrolytes for EES devices could repair the electrode/device fracture and mitigate the deformation damage, extending the lifetime of EES devices. Although significant progress has been made in recent years, the development of functional electrolytes for smart EES devices is still in its infancy stage with many challenges and opportunities to be addressed in the future.

One important concern lies in the unsatisfactory electrochemical properties of currently developed functional electrolytes. Most of the aforementioned functional electrolytes for EES devices are still based on proof‐of‐concept studies. The reported electrochemical performances of EES devices using functional electrolytes are much lower than those of EES devices based on conventional electrolytes. In many cases, the introduction of smart functions has sacrificed the ionic conductivity and voltage window of the electrolytes. In future research, smart functions and basic electrolyte properties need to be compromised to reach the optimal balance. Moreover, it is necessary to develop functional electrolytes that respond to multiple external stimuli. Integration of multiple functions into electrolytes is vital to satisfy the demands of EES devices under complex conditions.

At the device level, the smart designs of functional electrolytes may deteriorate the power densities of EES devices to some extent. This is possibly because the introduced smart materials in functional electrolytes lead to the significant increase in the interfacial charge transfer resistance of electrode materials. The side reactions and interfaces between functional electrolytes and electrodes have not yet been well studied. It is highly desirable to investigate the side reaction and functional electrolyte/electrode interface evolution by various in situ electrochemical spectroscopy or microscopy characterizations and advanced theoretical simulations.

In addition, all smart EES devices using the aforementioned functional electrolytes are still limited to laboratory research, far away from large‐scale production. Different from currently commercial EES devices with conventional liquid electrolytes, smart EES devices may have different device configurations. For example, EES devices using sunlight‐responsive electrolytes and electrochromic electrolytes require transparent sealing packs. The challenge is even greater in the practical application of smart EES devices owing to the requirements of synchronous optimization of electrode materials, functional electrolytes, and sealing packs. The good processability, long stability, and compatibility of all components are all essential to the successful application of functional electrolytes in EES devices.

## Conflict of Interest

The authors declare no conflict of interest.
